# Hospital Meetings

**Published:** 1901-12-21

**Authors:** 


### HOSPITAL MEETINGS.

LEAGUE OF MERCY: SECOND ANNUAL MEETING.

The Presidents of the League of Mercy held the second annual meeting on Monday afternoon in the Governors' Court Room, Guy's Hospital, Sir Whittaker Ellis, Bart., in the chair.

There were present the Marquess Camden, the Earl of Mansfield, Lord Pirbright, Lady Pirbright, Lord Farquhar, K.C.V.O., Lady Florence Pelham Clinton, Lady Edith King-Noel, the Hon. Mrs. Freeman-Thomas, Sir J. Whittaker Ellis, Bart., Sir Fitzroy Maclean, Bart., C.B., Lady Faudel-Phillips, Lady Burdett, Col. Campbell, Mrs. Seymour Corkran, Alderman John Harris, Mr. C. Tyrrell Giles, Mrs. Tyrrell Giles, Capt. Gunton, Mr. Montagu Sharpe, Mrs. R. L. Harrison, Col. A. A. Owen, Mrs. Sidney Straus, Mr. B. S. Straus, Mr. A. H. Tarleton, Mrs. E. J. Sanders, Mrs. Lewis, Miss Harington, Col. Edwin Hughes, M.P., Mrs. Hughes, Mr. F. S. W. Cornwallis, Mr. Ambrose Pomeroy, Mr. H. W. Keay, Mrs. Buxton Morrish, Mrs. Wightman, Mr. E. E. Pearson, Mr. T. W. Idris, Mr. James Round, M.P., Sir Henry Burdett, K.C.B., Treasurer; Lord Wolverton, Dr. W. J. Collins, and Mr. J. Harrison, Hon. Secretaries to the League; Mr. N. Hay-Forbes, F.R.C.S.Ed., Hon. Secretary to the Presidents.

After the minutes of the last annual meeting had been read and confirmed, a letter was read from Sir Francis Knollys stating that their Royal Highnesses the Prince and Princess of Wales had graciously consented to accept the offices of Grand President and Lady Grand President of the League. The Chairman, in expressing the gratification of the League, said he could hardly do so without referring to the recent tour of the Prince and Princess round the world, because that tour affected the work in which the League was engaged. England was noted for her charities to the sick, the poor, the stranger, and those from whom she differed in opinions; and it might fairly be hoped that the visit of their Royal Highnesses to his Majesty's Dominions beyond the Seas had opened the door to similar charitable feelings in the colonies. Nothing could be more likely to bring this about than the voyage which had been looked upon throughout the world as one of the most glorious ever undertaken by Royal personages. This expression of gratification, having been seconded by the Marquis Camden, was carried unanimously.

The election of the Honorary Chairman, Deputy Chairman, and Secretary to the President for the forthcoming year was proposed by Mr. Montagu Sharpe as follows: Chairman, Sir Whittaker Ellis; Deputy Chairman, Mr. E. A. Hambro; Hon. Secretary, Mr. Norman Hay-Forbes, F.R.C.S.Ed. This was seconded by Lord Wolverton and carried unanimously.

The Chairman said the task the League had to perform was rather a difficult one, but those connected with it had laboured in the past, and would do so again. He believed that the difficulty lay in the fact that the working classes had been so long accustomed to receive everything without themselves contributing towards the cost that it was extremely difficult to imbue them with the idea that they could help by the small contributions they were able to make. He thought it was Bacon who had said that "the cup must be full before it could run over"; it was perhaps because the working man's cup was not full that subscriptions did not come from him, for when rent had been paid, and provision for the family made, there was not much left. There were, however, millions who could give, say, a penny a month, and it was the duty of the League to try and induce them to see the benefits of helping others, even by small contributions.

The representatives to the Executive Council having been appointed for the year, Sir Henry Burdett said he was very pleased to announce that, in spite of difficulties—he thought even greater difficulties than last year—the actual sum received exceeded that of last year by some £300. Last year was difficult because it was the first year after the first flush of enthusiasm, when those who had taken office began to realise that they had a difficult task, which could only be rendered successful by a great amount of devoted individual service. That the League had survived its second year without altering its *personnel* was a very good sign. He thought the League was now fairly started, with a band of zealous helpers, who were doing increasingly useful work, and those districts that were doing exceptionally good work were an encouragement to those not doing so much. They were proving that small subscriptions could be raised, and this success would, he thought, attract some who had not yet come forward. As the returns stood at present, Sevenoaks stood highest, with upwards of £600; next came St. George's, Hanover Square, with £450 16s. 6d.; Fulham had sent £449 13s. 6d., and Kingston came fourth with £441 11s. One of the most encouraging points about this return was the increase of subscriptions from the home counties, so much so that he thought the notion that they would not do much was dying out. Men and women were finding out the worth of personal service; and on behalf of the Executive Committee he heartily thanked the Presidents for their labour and zeal. He hoped the results would encourage them to thank God and take courage to go forward. In reply to a question as to the recognition of the services of those Vice-Presidents who, devoting their energies to collecting for local hospitals, could not also collect for the League, Sir Henry Burdett said that the record of exceptional service would probably reach the ears of those interested in hospitals, but that such services could not be included in the reports of the League. In connection with the Coronation gift, he explained that the effort that was being made by the promoters of the gift was something altogether apart from the work of any other fund, and that it was moreover a temporary effort; he hoped no one would think that by subscribing to that gift they were entitled to withhold their usual subscriptions from any other fund. This, in his opinion, would be inconsistent with good citizenship.

In reply to the question why the report had only been sent to those who had sent in subscriptions, and not to all Vice-Presidents, the Earl of MANSFIELD thought it would have been advantageous if the report for last year had been sent to all Vice-Presidents, even those who had collected nothing.

Mr. HARRISON (Hon. Secretary) said that the report had been sent to every Vice-President who had obtained anything, however small, but that a large list of Vice-Presidents had been received, from 1899 onwards, of whom nothing further had been heard, and it was thought absolutely useless to send them reports. Reports, however, were always sent whenever they were asked for.

Votes of thanks to the authorities of Guy's Hospital for the use of the room was moved by Lord PIRBRIGHT, seconded by Colonel CAMPBELL, and carried unanimously; and a vote of thanks, moved by Lord FARQUHAR, to Sir Whittaker Ellis brought the meeting to a close.

The authorities of the hospital entertained the Presidents and Lady Presidents at tea, and invited them to visit the hospital; an invitation which was much appreciated and taken advantage of.

### HOSPITAL SUNDAY FUND: ANNUAL MEETING.

THE LORD MAYOR presided at the annual general meeting of the constituents of the Metropolitan Hospital Sunday Fund, at the Guildhall, on Monday (16th inst.) at noon. The report for 1901 was received and adopted, on the motion of the Rev. C. H. GRUNDY, and seconded by the Rev. Dr. RIGG. Both speakers alluded to the difficulties of the year, but thought the promoters of the Fund should feel encouraged to hope for greater success next year. Mr. Grundy said it had been thought that the events in connection with the Coronation might do harm to the Fund, but he hoped, on the contrary, that their receipts would be doubled. He suggested that the laity should stir up the clergy to fresh efforts.

In reply to a speaker from the body of the hall, who asked whether money given by the Fund had been diverted from the hospital wards to the medical schools, as had been the case with the Prince of Wales' Fund, Sir SAVILE CROSSLEY said he rose to a point of order. He had the honour to be Honorary Secretary of the Prince of Wales' Fund, and he wished to say that no money had been given by that Fund to the medical schools.

The LORD MAYOR said the Hospital Sunday Fund had nothing whatever to do with the matter; it was not their province to inquire before allocating the funds—which were given for the grand whole—whether the hospital to which they gave the grant were assisting its school or bringing the school up to the standard of modern requirements. In giving the money they were doing their duty by this generation and those to come.

Sir EDMUND HAY CURRIE in proposing that the laws of the constitution, which have been in force during the last year, be continued, alluded to the ill-health of the secretary, Mr. Henry N. Custance, who had only missed one annual meeting during the whole time of the Fund's twenty-nine years of existence. He would like Mr. Custance to know that the constituents sympathised with him in his illness, and that they appreciated his unfailing interest and conscientiousness in his work; he hoped that the leave which had been granted him until the spring would restore him to health. On the seconding of Sir SAVILE CROSSLEY, the resolution was carried.

The Council having been re-elected, with the Right Rev. the Bishop of Islington, the Honourable Rupert Guinness, C.M.G., Sir Frank Green, Bart., and J. T. Scriven, Esq., to fill vacancies, the Ven. Archdeacon SINCLAIR proposed "That June 15th be fixed for Hospital Sunday of 1902, and that the cordial co-operation of all ministers of religion within the Metropolitan area be again invited in the usual way."

The CHIEF RABBI said that the only year in which the receipts had exceeded last year's amount was the Jubilee year; he hoped that a similar stimulus would be given by the approaching Coronation; as the King's interest in hospitals was well known, there was every reason to hope that this would be the case.

In replying to a vote of thanks to the Chair, the LORD MAYOR said he had been connected with hospitals almost from a boy, and he hoped to do all that was possible for the Fund, taking into consideration his heavy duties, both civic and Parliamentary. The work of hospitals was not only in the wards, but also in the great schools attached, and for what was done there the generations to come would bless the hospitals.

